# New era of Genes and Environment

**DOI:** 10.1186/s41021-015-0005-9

**Published:** 2015-06-16

**Authors:** Yasunobu Aoki

**Affiliations:** Center for Environmental Risk Research, National Institute for Environmental Studies, 16-2 Onogawa, Tsukuba, Ibaraki 305-8506 Japan

**Keywords:** Environmental agent, Genotoxicity, Mutagen, Mutation research, Open access Journal

## Abstract

Genes and Environment is now at the start of a new era of publication as an open access journal. I believe that open access publication marks a turning point that will make *Genes and Environment* unique in the Asian environmental mutagen research community.

In 2015 *Genes and Environment* is at the start of a new era of publication as an open access journal. The transformation of *Genes and Environment*, the official journal of the Japanese Environmental Mutagen Society (JEMS), to open access is the beginning of a great epoch for JEMS. I would like to especially thank Dr. Takashi Yagi Editor-in-Chief, for his tireless efforts in this process, which was planned in May 2014 by the Editorial Committee. After discussions among Editorial Committee members and Council members, JEMS chose BioMed Central as the journal’s open access publisher. Articles in *Genes and Environment* are now accessible via the journal’s homepage at BioMed Central, and previous content can be accessed on the JEMS homepage.

*Genes and Environment* has published many outstanding articles in the field of mutation research, including articles on the mechanisms by which environmental agents induce mutations. I believe that open access publication marks a turning point that will make *Genes and Environment* unique in the Asian environmental mutagen research community. Asian countries share issues that need to be resolved; for example, the people of Asia are concerned that diseases such as cancers may be caused by agents in environmental media such as the air or water, or in food, and this concern will be an important topic in the new *Genes and Environment*.

JEMS plans for growing *Genes and Environment* into a mainstream journal in the field of mutation research. All mutation researchers are invited to submit their latest manuscripts to *Genes and Environment*.

